# Is Oxford Nanopore sequencing ready for analyzing complex microbiomes?

**DOI:** 10.1093/femsec/fiab001

**Published:** 2021-01-14

**Authors:** Lee J Kerkhof

**Affiliations:** Department of Marine and Coastal Sciences, Rutgers, the State University of New Jersey, New Brunswick, NJ 08901, USA

**Keywords:** MinION sequencing, microbiome, 16S rRNA gene, antibiotic resistance gene, ribosomal RNA operon

## Abstract

This minireview will discuss the improvements in Oxford Nanopore (Oxford; sequencing technology that make the MinION a viable platform for microbial ecology studies. Specific issues being addressed are the increase in sequence accuracy from 65 to 96.5% during the last 5 years, the ability to obtain a quantifiable/predictive signal from the MinION with respect to target molecule abundance, simple-to-use GUI-based pathways for data analysis and the modest additional equipment needs for sequencing in the field. Coupling these recent improvements with the low capital costs for equipment and the reasonable per sample cost makes MinION sequencing an attractive option for virtually any laboratory.

## Teaser

The Oxford Nanopore MinION is the preferred platform for studies in microbial ecology, because of its long-read capability, low cost and ease of use.

## INTRODUCTION

Nucleic acid-based methods investigating ribosomal RNA genes have become the most widely accepted way to characterize microbial communities in the last 35 years. Initially, a clone and sequence approach involving 5S and 16S rRNA genes was employed (e.g. Stahl *et al*. 1985; Olsen *et al*. 1986). Subsequently, ribosomal RNA gene data were collected using second-generation platforms, focusing on variable regions within the 16S rRNA gene due to the ability to rapidly generate sequence reads compared with traditional Sanger methods (e.g. Illumina, pyrosequencing; Sogin *et al*. 2006; Roesch *et al*. 2007; Lazarevic *et al*. 2009; Whiteley *et al*. 2012). These second-generation approaches often provided large numbers of short reads (<500 bp) with high accuracy (∼99%), but limited phylogenetic resolution—rarely below genus level due to the short-read length and highly conserved nature of the target gene. However, within the last 5 years, a third-generation system for direct, long-read sequencing of individual strands of DNA has become available, principally the Pacific Biosciences of California (California, US; PacBio) and Oxford Nanopore Technologies (Oxford, UK; ONT) systems.

This mini review will be focused on the ONT MinION as a platform for microbiome analysis due to its low cost and portability. Recent summaries of capital/reagent costs indicate most second-generation sequencing platforms and the PacBio system range from $80 000 to $800 000 (Loman *et al*. 2012; Quail *et al*. 2012). In contrast, the ONT MinION is available for $1000, but requires a separate computer for data collection (bringing the capital cost to $3500–$5000). A new version of the ONT sequencer [the MK1C] combines the computer, a touch screen, 1 TB SSD drive, a 6-core CPU and a 256-core GPU in a single, handheld unit for the same capital costs. Nanopore sequencing is low cost because it is predicated on measuring the electrical conductivity of individual DNA strands translocating protein pores in a semiconductor membrane (Schneider and Dekker 2012). For the ONT system, each DNA molecule has an adaptor ligated to one end that interacts with a docking protein and binds to the nanopore. This docking protein regulates the speed by which the DNA molecule traverses the membrane (initially 50 bp/s; now 450 bp/s for each active pore). The DNA sequence is then determined on 5 bp segments (*k*-mers) by measuring the change in electrical conductivity across the membrane as the DNA flows along each individual channel. The basecalling is done using hidden Markov or recurrent neural network models to convert the electrical ‘squiggles’ into the various nucleotides.

Overall costs to perform a MinION sequencing run are modest. For example, a MinION flow cell costs $900 (if bought individually), a sequence reaction is $100, while additional enzymes/reagents for PCR/end-repair/library preparation/washing are ∼$200. If 40 barcodes are used per sequence run and each flow cell can be used at least twice (with washing), costs are ∼$1200 per 80 samples, which comes to $15 per sample. Furthermore, the newer MinION chemistries are providing >2 million reads within 24 h in many laboratories. This yield is roughly 50 000 raw reads per sample, providing up to 30 Gb per flow cell. Given the cost of the hardware is small, the required reagents are modest and a service contract or dedicated technician is not required, the MinION system is the first widely affordable sequencing system for virtually any laboratory.

However, major questions remain regarding the suitability of this platform for microbiome profiling, including:

- Does the MinION have too high an error rate that would prevent accurate microbial community analysis?
- Can MinION provide a quantitative signal that reflects target abundances within the original sample?
- Can existing or simplified data analysis pipelines be utilized with MinION?
- What additional equipment is needed for MinION use in the field?

Here, I will attempt to show that the ONT MinION is actually the preferred platform for studies in microbial ecology, because of its long-read capability, low cost and ease of use.

## IMPROVING SEQUENCE ACCURACY

Since the ONT MinION was commercially introduced in 2014/2015, major strides in both read quantity and accuracy have been achieved. These milestones have resulted from improvements in sequencing chemistry/pore design as well as better algorithms for basecalling. As a result, sequence read accuracy has increased during this period. A more complete synopsis of the improvements in chemistry and basecalling accuracy is provided by Rang, Kloosterman and de Ridder (2018). In their review, the timeline of changes from R6 to R9 chemistry [SQK-MAP006 to SQK-LSK009] and the shift from basecalling using the MinION read-capture software (MinKNOW) to an offline-capable, basecaller (Albacore 2.0) is well documented. The improvements in sequence accuracy from 60 to 90% are illustrated during 2014–2018. Interested readers are encouraged to read this review and others like it.

Most of the published MinION scientific studies utilize earlier versions of the MinION basecaller. However, in late 2018, there was a shift by ONT to the Guppy algorithm (v2.0), a recurrent neural network basecaller. Upon Guppy release, ONT indicated a median read accuracy of 89–94% from four microbial genomes (*H. pylori*, *M. maripaludis*, *P. acnes* and *T. thermophilus*), while Wick *et al*. (2019) found that Guppy 2.2.3 could provide a read accuracy of 87–89% when re-sequencing a *Klebsiella pneumoniae* isolate. There have been multiple updates to the Guppy algorithm in the last 18 months. Furthermore, at the London Calling meeting of 2020 (an annual showcase of ONT research by investigators from around the world), the median single read accuracy for Guppy 3.6.0 when sequencing a mixture of reference microbial genomes or the human genome was reported at 96.5%. The current version of Guppy (4.0) was released in June 2020. Importantly, because long-read technology measures the electrical signal as a DNA molecule transits a nanopore, the ability to re-analyze MinION data with newer algorithms and improve sequence accuracy on completed runs becomes possible. This is either a major problem or a strength, depending on your perspective. It can be disconcerting that MinION sequences are not fixed once the analysis is complete and can be changed depending on the basecalling algorithm. On the other hand, re-analysis of MinION reads may provide an avenue for genome closure or single nucleotide polymorphism (SNP) detection with improved sequence accuracy that is not possible using sequence-by-synthesis methods.

Another traditional approach to improve sequence accuracy is by generating a consensus sequence. During the time of Sanger sequencing, it was mandatory to read both DNA strands before depositing any consensus sequence in GenBank. In one of the earliest studies on 16S rRNA genes using MinION, Benitez-Paez, Portune and Sanz (2016) analyzed a 20-member mock community by sequencing an ∼1400 bp amplicon. The authors reported being able, ‘to reconstruct nearly full-length16S rDNA sequences for [the] 20 different species analyzed from the mock bacterial community and demonstrated a consensus accuracy of 92–94% using MinKNOW basecalling of R6 kit-2D reads'. They reported ‘an acceptable taxonomy assignation…..only limited by the sequencing effort’. Since then, Li *et al*. (2016) using a three-member system developed a rolling circle amplification/adaptor-directed consensus building method termed "Intramolecular-ligated Nanopore Consensus Sequencing (INC-Seq) and compared PacBio and MinION platforms. These authors also used R6 chemistry, MinKNOW basecalling and three sets of 500 bp windows to define similar reads for consensus building. The authors found that INC-Seq improved the median read accuracy on both the PacBio and MinION platforms to nearly the same extent (84–98% for PacBio; 84–97% for MinION) when employing between 6 and 15 copies for the consensus. Subsequently, Calus, Ijaz and Pinto (2018) developed additional consensus building steps for the INC-Seq pipeline, including chopSeq for uniform read alignment and nanoClust for partitioning, to identify reads for consensus building in a *de novo* manner. By employing R8 chemistries with Albacore 1.2.4 basecalling, the authors could generate near-full length 16S rRNA genes with mean sequence accuracies >99% when building a consensus from 3 to 50 reads. Another development for generating consensus sequences for rRNA genes is reported by Karst *et al*. (2018), where the researchers incorporated unique molecular tags prior to sequencing and generated >10 000 copies of each amplicon for consensus building. Most of their study involved Illumina sequencing of SSU genes recovered via polyA tailing rather than traditional PCR primers. However, the authors indicate this approach also worked for MinION platform and reduced the mean read error rate from 10 to 1% with R7 and MinKNOW basecalling (Supp. Fig 8 in citation).

Along similar lines, the analysis of near-full length ribosomal operons (4–6 Kb) is now possible with the ONT MinION. These rRNA operons are amplified using domain-specific forward primers in the small ribosomal RNA subunit (16S), domain-specific reverse primers within the large rRNA subunit (23S) and a long-range, high-fidelity Taq polymerase. The rRNA operon profiling approach yields an amplicon containing both 16S/23S ribosomal subunits for phylogenetic assignment (4200 bp of sequence), plus the ITS region (500+ bp) containing species/strain information to distinguish various members within the microbial community. The first reports of rRNA operon sequencing using the MinION utilized two different mock communities (Benitez-Paez and Sanz 2017). These researchers did not attempt to build a consensus from their MinION reads. Rather, the authors created an rRNA operon database of ∼22 000 entries, investigated the accuracy of 1D reads from R6; R8 chemistries/R9; R9.4 flow cells, and compared their MinION results with Illumina MiSeq V4–V5 methods. The authors found rRNA operon matches to bacterial species in their database for 16 of 20 members of 1 mock community and 8 of 10 in the other. In contrast, the MiSeq data could only resolve to the genus level with a comparable coverage. Additionally, the authors report improvements in MinION median read accuracy from 69 to 85% for the different chemistries with the maximum read accuracy increasing from 87 to 92%. Using a similar approach, my lab employed the MinION to analyze ribosomal operons from complex, environmental samples with R6 chemistry and MinKNOW basecalling (Kerkhof *et al*. 2017). Replicate rRNA operon PCR products from known mixtures of farm soil and NASA bioreactor DNA were barcoded and sequenced. This approach detected over 1000 different ribosomal operons, each uniquely matching entries from the NCBI 16S rRNA gene database. Those 2D reads with >35x coverage were then used to reconstruct 30 rRNA operons in an iterative/bootstrap fashion via LastZ alignment. The results yielded ribosomal operons with 16S rRNA sequences matching the Proteobacteria, Actinobacteria, Acidobacteria, Firmicutes and Gemmatimonadetes with 92 ± 5% identity to the NCBI database (Table 1 in citation). Phylogenetic analysis of the 16S rRNA and 23S rRNA genes from each operon demonstrated nearly identical tree topologies with species/strain level resolution and no detectable chimera formation.

**Table 1.** List of classifier, target gene, sample (mock community or complex environmental), database and citation for MinION studies investigating microbial systems. ‘X’ indicates yes for column (i.e. analyzed a mock community?) or a linear correlation coefficient >0.69. ‘O’ indicates a linear correlation coefficient <0.25.

Classifier	Sequence	Mock	Complex sample	GUI	Quantifiable	Database	Reference
BLASR, LAST	16S rRNA gene	X				Self reference	Kilianski *et al*. 2015
BLAST-Discontiguous MegaBlast	rRNA operon		Farm soil, bioreactor	X	X	NCBI 16S rRNA	Kerkhof *et al*. 2017
BLAST-Discontiguous MegaBlast	rRNA operon		Human respiratory samples	X	X	EZ BioCloud	Ibironke *et al*. 2020
BLAST-Discontiguous MegaBlast	rRNA operon		Mouse feces	X		NCBI 16S rRNA	Dowden *et al*. 2020
BLASTn, Mothur	16S rRNA gene	X		X		Self reference	Calus, Ijaz and Pinto 2018
BLASTn	16S rRNA gene	X		X	X	Customized SILVA DB	Li *et al*. 2016
BLASTn, Centrifuge	16S rRNA gene	X	Human pleural biopsy	X		NCBI RefSeq, GenomeSync	Mitsuhashi *et al*. 2017
BLASTn, MG-RAST	Genomic DNA		Arctic soil/isolates	X		NCBI nr/nt, MG-RAST	Goordial *et al*. 2017
BLASTn, minimap2	Genomic DNA	X	Human neonate feces	X	X	NCBI nr/nt, Self reference	Leggett *et al*. 2020
BLASTp	Genomic DNA		*Caulerpa ashmeadii* holobiont	X		NCBI nr/nt	Sauvage *et al*. 2019
BLAST2GO	16S rRNA gene		Coastal seawater	X		NCBI nr/nt	Curren *et al*. 2019
Burrows–Wheeler Aligner	Genomic DNA	X			O	Self reference	Sevim *et al*. 2019
Centrifuge	18S-ITS-28S	X	Coastal seawater			Self reference	Hatfield *et al*. 2020
Centrifuge, Kraken, Kraken2,	Genomic DNA	X			X	Precompiled DB	Leidenfrost *et al*. 2020
Centrifuge, Kraken, WIMP	Genomic DNA	X		X		NCBI RefSeq	Deshpande *et al*. 2019
Centrifuge, Kraken2	REP-PCR		Isolates			Precompiled DB	Krych *et al*.^2019^
CosmID, MG-RAST	Genomic DNA		Riverine samples			Proprietary DB, MG-RAST	Hamner *et al*. 2019
EPI2ME	16S rRNA gene	X	Fresh water samples	X		NCBI RefSeq	Acharya *et al*.^2019^
EPI2ME	16S rRNA gene		Human cerebral/spinal fluid			NCBI RefSeq	Hong *et al*.^2020^
EPI2ME	Genomic DNA	X	Human respiratory samples	X	X	NCBI RefSeq, ARMA	Yang *et al*.^2019^
EPI2ME, GraphMap, Mothur	16S rRNA gene	X		X		NCBI 16S rRNA	Winand *et al*. 2020
Epi2ME, Kraken, MG-RAST, One Codex	Genomic DNA	X			O	NCBI RefSeq	Brown *et al*. 2017
EPI2ME, minimap2	rRNA operon	X	Dog skin	X	X	NCBI RefSeq, rRNA operon DB	Cuscó *et al*. 2019
EPI2ME, Centrifuge	Genomic DNA		Swabs from oil paintings	X		NCBI RefSeq	Pinar *et al*. 2020
GenomeSync Tool Kit, custom script	16S rRNA gene	X			O	GenomeSync DB, NCBI DB	Kai *et al*. 2019
Kraken2, MG-RAST, One Codex	Genomic DNA		Riverine samples			Custom reference DB	Reddington *et al*. 2020
LAST	16S rRNA gene		Mouse feces			GreenGenes DB	Shin *et al*. 2016
LAST	16S rRNA gene	X	*Alexandrium tamarens* phylosphere		X	SILVA DB	Shin *et al*.^2018^
LAST	rRNA operon	X				rRNA operon DB	Benitez-Paez and Sanz 2017
LAST, Centrifuge	Genomic DNA		Wastewater		X	SARG DB, NCBI nr/nt DB	Che *et al*.^2019^
LAST,QIIME	16S rRNA gene, HPV gene		Human cervical sample			GreenGenes DB	Quan *et al*. 2019
Metapathways, MG-RAST	Genomic DNA		Polar sediments, cultures			NCBI 16S rRNA, EZ BioCloud	Millan-Aguinaga *et al*. 2019
MG-RAST	Genomic DNA		Riverine sediments			MG-RAST DB	Samson *et al*. 2019
RDP Classifer, QIIME	16S rRNA gene		Emergency medical service vehicles			GreenGenes DB	Sheahan *et al*. 2019
SINA aligner	16S rRNA gene	X				SILVA DB	Benitez-Paez, Portune and Sanz 2016
Usearch	Genomic DNA		Stormwater			Custom Human Gut DB	Hu *et al*. 2018

Finally, similar consensus building approaches for genome assembly have also been employed with MinION reads and multi-x coverage for removing errors. One of the earlier examples of complete genomes using Nanopore sequence data was for *E. coli* K12 with 99.5% identity (Loman, Quick and Simpson 2015) and ∼29× coverage and 99.8% identity for *Francisella* strains (Karlsson *et al*. 2015) with 30–60× coverage. Another approach to complete genome closure combines Nanopore and Illumina reads to create a synthetic consensus for assembly/alignment (NaS fragments up to 60 kb in length) with 99.99% accuracy for *Acinetobacter baylyi* (Madoui *et al*. 2015) with 34–50× coverage. Since then, other hybrid programs have been developed, such as Unicycler, which uses both long and short reads to resolve conflicts and provides longer, high-quality assemblies than other programs (Wick *et al*. 2017). Wick *et al*. (2019) used their assembler and various ONT basecallers in their testing of genome reconstruction for *Klebsiella pneumoniae* at 100× coverage. The authors determined a consensus accuracy of 99.40–99.85% could be achieved, which could be improved to 99.94% by employing a custom training set for basecalling rather than the general ONT parameters. The researchers concluded that very noisy individual reads can provide an accurate consensus if the sequence errors are randomly distributed and there is sufficient coverage which is supported by the other studies cited above.

## QUANTITATIVE ABILITY OF THE MINION

To date, much of the research testing the ability of MinION to identify microbes has utilized commercially available mock communities containing either equal molar or varying target concentrations with up to 20 members (Kilianski *et al*. 2015; Mitsuhashi *et al*. 2017; Calus, Ijaz and Pinto 2018; Acharya *et al*. 2019; Hatfield *et al*. 2020; Winand *et al*. 2020). Unfortunately, most of these studies have been focused on detection by the MinION rather than a quantitative response. However, in early work by Benitez-Paez, Portune and Sanz (2016) using the 16S rRNA gene and the equal molar BEI mock community, the authors reported 2–4-fold variations in read numbers for the various SSU genes compared with the actual target abundance within the sample. They also found a strong correlation between the coverage deviation and the calculated number of rRNA operons for three members of the community. The quantitative response of their MinION study for the remaining members of the mock community was not tested. Along similar lines, Brown *et al*. (2017) investigated both a 4-member and a staggered concentration 20-member mock community (HM-783D) by directly sequencing genomic DNA and analyzing via three classifiers [MG-RAST, What's in my Pot (WIMP; now part of EPI2ME) and One Codex]. These researchers were also the first to enhance the input genomic DNA signal by φX amplification (GenomoPhi). The authors describe read numbers within 10% abundance of the mock community abundance for unamplified target. However, a strong bias was observed with genome amplification (*r*^2^ < 0.2 from Table 4 in citation). Subsequently, Kai *et al*. (2019) investigated both genomic DNA extracts and a whole cell mixture of a 10-member mock community using a newly available16S rRNA gene sequencing kit from ONT (R9 chemistry with improved accuracy). The study was designed to determine if directly amplifying SSU genes from bacterial biomass could be a viable option for medical diagnosis by testing a direct cell lysis, a bead beating step and a more traditional DNA purification. Their efforts were also focused on rapid detection via MinION at 3, 5 and 30 min intervals. Although a histogram is presented of MinION reads with largely similar relative percentages to the mock community for the different time points, the deviation from the actual abundance in the mock community was sufficient to have an *r*^2^ < 0.1 (from Fig. 5 in citation). Likewise, Sevim *et al*. (2019) compared the read response for total genomic DNA sequences by Illumina, PacBio and MinION from a 12-member mock community and found a correlation of *r*^2^ <0.24 with target dosage for all three platforms (Table S2 in citation).

In contrast, the INC-Seq (rolling circle amplification) approach by Li *et al*. (2016) also tested a 10-member mock community and had a much better adherence of MinION reads to the original dosage in the sample. The linear regression of the relative percentage data from Table 3 in the citation is *r*^2^ = 0.69. However, if the highest data point representing over-amplification of the largest template in the mixture is removed, the *r*^2^ = 0.96. Leidenfrost *et al*. (2020) utilized a cocktail of 12 different bacterial genomic DNAs that were sequenced and analyzed by Kraken, Kraken 2 and Centrifuge. The number of reads assigned to each genome correlated well with the abundance in the mixture depending on the DNA quantitation method (*r*^2^ = 0.81 for quantitation by ddPCR and *r*^2^ = 0.97 for quantitation by Qubit; Table S5 in citation).

**Figure 1.**
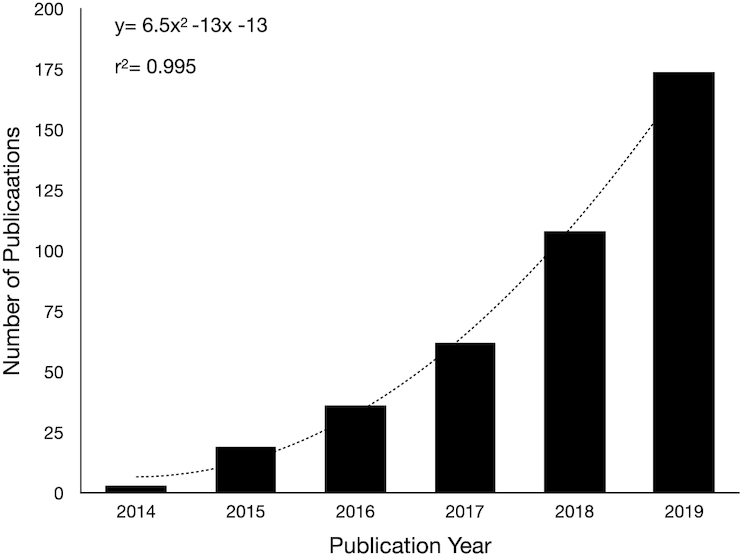
Plot of publications in Web of Science using the search term ‘MinION’ from 2015 to 2019.

Similar quantitative results have also been obtained using entire ribosomal operons. As stated above, my lab created DNA mixtures ranging from 10 to 100% abundance using two end-member communities (i.e. farm soil/bioreactor DNA; Kerkhof *et al*. 2017). We assessed the ability of MinION to quantify the most abundant best BLAST hits from quadruple amplifications/sequencing runs. All of the reads associated with the top OTUs (10% of total OTUs detected) responded in a quantitative fashion with an *r*^2^ averaging 0.82 ± 0.14 (*n* = 104 unique BLAST hits to the NCBI 16S rRNA database; Fig. S7 in citation). Cuscó *et al*. (2019) also utilized rRNA operons and SSU genes to assess a mock community and the microbiome from the skin of dogs on the chin and back. The authors report robust quantitative response by 16S rRNA genes to dosage in the mock community (*r*^2^ = 0.95; Fig. 3 in citation), with a slightly lower correlation coefficient for the rRNA operons (*r*^2^ = 0.82). Finally, Leggett *et al*. (2020) investigated a 20-member mock community before assessing the fecal microbiome in human neonates by total genome analysis on the Illumina and MinION platforms. They found a robust relationship between log transformed read number and dosage for both sequencing platforms (Pearson's coefficient for MinION, *r* = 0.94; for Illumina, *r* = 0.97; Fig. 1 in citation). From all the above studies, it is clear that the MinION has the potential to provide quantitative information on the microbiome from complex environmental samples. However, given the large differences in DNA quantitation methods, PCR conditions/primers, sequencing chemistries and classification software (e.g. Centrifuge, BLAST, LAST, etc.), it is prudent for each researcher to empirically demonstrate a predictive/quantitative response for MinION reads and gene dosage in their particular system.

### Can existing or simplified data analysis pipelines be utilized with MinION?

The different software algorithms for analyzing MinION data have become nearly as varied as the researchers utilizing the platform and the samples being analyzed. Reviews by Leggett and Clark (2017) and Magi *et al*. (2018) detail the transition in data capture/basecalling software by Oxford Nanopore (MinKNOW and Metrichor) to Albacore as well as the various polishing, demultiplexing and assembly software created by independent researchers to improve nanopore sequence accuracy and genome reconstruction. Interested readers are encouraged to read these reviews. What has not been as widely documented is the various classifying algorithms used to determine the composition of the microbiome. Table 1 provides an overview of the various classifiers and databases employed in over 30 studies in mock or complex, environmental communities with the MinION. Both BLAST and Centrifuge are the most utilized algorithms, partly because these classifiers are part of the EPI2ME data analysis package with Oxford Nanopore. The EPI2ME software is a simple, graphical user interface that screens MinION fastq reads against the NCBI RefSeq, NCBI 16S rRNA or the Comprehensive Antibiotic Resistance Database (CARD). The user has the ability to select multiple analyses, including demultiplexing of ONT barcodes, taxonomic assignment by BLAST of 16S rRNA genes, determination of ‘What's in My Pot (WIMP)' by Centrifuge alignment or antibiotic resistance mapping by minimap2. Input includes both amplicon and genomic DNA sequences with the output providing a frequency summary of taxonomic assignments of reads, phylogenetic placement within an NCBI taxonomic tree and an exportable summary table of results as CSV/TSV files. Additional information includes a description of taxa from Wikipedia and the CARD resistance ontology. The user has the option of varying the display of phylogenetic resolution by clicking on different nodes within the tree and exporting the graphical output as a png file. Another simple, alternative GUI has been developed, called MINDS, which is capable of offline screening of MinION reads with Centrifuge. The software was designed for users without a scientific education or laboratory background, with a focus on detection and enumeration (Deshpande *et al*. 2019).

The other most common classifier for MinION sequences is the BLAST algorithm. Both BLASTn and Discontiguous MegaBLAST have been used to assign MinION ribosomal RNA reads to taxa using customized databases (*n* < 100), the NCBI 16S rRNA gene database (now containing *n*> 21 500 entries) or the EZ BioCloud database (*n* > 61 000 entries). Genomic sequence reads from the MinION have also been aligned against the NCBI RefSeq (*n*> 35 million sequences) or the nr/nt database (*n* > 60 million sequences). Unfortunately, many researchers do not report the various BLASTn parameters that were employed when analyzing their MinION data. However, the BLASTn default parameters are a word search length of 11, gap cost of 5/2 and match/mismatch scoring of 2/−3. Interestingly, these same parameters were found to correctly identify near-full length 16S rRNA genes that had been mutated *in silico* to resemble raw MinION reads (80–100% identity) at the species level from the NCBI 16S rRNA database with Discontiguous MegaBLAST (Kerkhof *et al*. 2017; Supp. Figs 5 and 9 in citation). A graphic-user-interface (GUI) for BLAST searches is available at the NCBI website and in many DNA analysis software packages (e.g. Geneious, BLAST2GO, Lasergene, etc.). Often, these GUI-based DNA software packages can also demultiplex, align and assemble MinION reads. The remaining classification platforms are not as user friendly and require a working knowledge of command line approaches. For example, the LAST and minimap2 classifiers are primarily used in the command line format. Command line Centrifuge and BLAST are also available and widely utilized to screen customized/indexed databases. There are also examples of researchers using well-established platforms such as QIIME or Mothur for MinION data analysis (Calus, Ijaz and Pinto 2018; Quan *et al*. 2019; Sheahan *et al*. 2019; Winand *et al*. 2020).

## ADDITIONAL EQUIPMENT REQUIRED FOR USE IN THE FIELD

One of the major attractions of the MinION is the portability. The MK1B fits in your hand and weighs <90 grams. Because the unit was designed to work with a laptop computer, the possibility of sequencing in the field can now be realized. However, a number of additional pieces of equipment are needed to process samples, prepare sequencing libraries and capture/process MinION data. One of the earliest reports of MinION use in the field involved epidemiology studies of Ebola virus in Guinea (Quick *et al*. 2016). These researches mobilized MinIONs, laptops, a PCR machine, heating blocks, pipettes and the reagents needed to begin sequencing within 2 days of arrival at Donka Hospital, Conakry, Guinea. All equipment could be carried in airport luggage and weighed <50 kg. The biggest issues these researchers faced were the need for uninterrupted power to run the MinION/laptop/PCR machines and trouble with internet connectivity. At the time of this study, the only pathway for sequence collection/analysis was MinKNOW that required real-time access to ONT servers. However, once routine internet connectivity could be established, Ebola sequence covering 97% of the genome was collected and analyzed offsite at servers in the UK. A similar field study of permafrost ice wedge soil and cryophilic isolates was undertaken with a portable MinION sequencing lab in the Canadian High Arctic the following year (Goordial *et al*. 2017). The authors needed internet access as well for basecalling and utilized a satellite internet link for one of their sequencing runs. However, they also had access to the first offline basecaller from ONT that was used for the three remaining sequencing runs in the field. The read data from isolates and the soil appeared to have been analyzed later on MG-RAST or NCBI servers. This approach has also been used to sequence in the dry valleys of Antarctica (Johnson *et al*. 2017). The primary objective was to see if the MinION could collect sequence under the harsh field conditions at the study site. As such, these authors constructed libraries at McMurdo Station and transported the MinION/sequence libraries to the Taylor Valley. They also had access to the offline version of MinKNOW from ONT for basecalling/data collection. The MinION was able to collect sequence data at temperatures down to −1°C and could be calibrated down to −5°C, although no sequence was collected at this low temperature. Similar approaches to test the ability of MinION to function under microgravity conditions have been done on the International Space Station (ISS; Castro-Wallace *et al*. 2017). In their first report, the authors utilized a Microsoft Surface Tablet rather than a laptop to run the MinKNOW software with the MinION. Data were collected on ISS and analyzed on the ground via servers or on a laptop computer. A subsequent study by Burton *et al*. (2020) demonstrated the ability to complete library prep/sequencing in microgravity, opening the door for microbiome analysis in space. Finally, one of the more recent examples of MinION sequencing in the field involves viral pathogen analysis of cassava fields in Sub-Saharan Africa (Boykin *et al*. 2019). Here, the researchers collected root/stem/leaf samples and the associated pests in Tanzania, Uganda and Kenya. Genomic DNA was extracted, sequenced and screened against a cassava mosaic disease database (CMD) on a laptop computer by BLASTn analysis using Geneious 11.1.2. The entire process (including sample processing, DNA sequencing and data analysis) was completed in <3 h at the farms. Essential equipment for the study is listed in Table 1 of their publication. The DNA purification was done using the PDQeX system (MicroGEM, New Zealand) powered by a 12 V lithium battery pack and the sequencing included a MinION connected to the ONT MinIT powered by a 20 000 mAH laptop power bank via a DC port at 20V. The MinIT is a stand-alone unit for MinION data collection/basecalling that functions without an internet connection and can transfer fastq files by WiFi for further analysis. This study is one of the first instances of a complete cycle of sequence analysis (DNA purification, data collection and read classification) in the field, using a fully offline platform. Most of the equipment outlined in Table 1 can fit into a backpack. The major limitations appear to be the ability to recharge the power banks and the necessity of having a well-defined database for classification. As such, new and emerging pathogens might need to be detected after returning to the laboratory. Finally, stand-alone software packages, such as SqueezeMeta, have been designed for metagenomic analysis, which run without high-performance computing infrastructure and in absence of any network connectivity (Tamames and Puente-Sanchez 2019). These authors could analyze 40 million reads on a standard laptop computer (8 cores, 16 GB RAM) in 10 h, generating 33 660 contigs in 38 bins and >124 000 functionally and taxonomically annotated genes.

### Examples of microbiomes/metagenomes characterized using MinION

Since the use of MinION has been gaining wider acceptance, the number of publications has been growing exponentially (Fig. 1) and the types of complex samples being analyzed have also expanded. In one of the earliest reports of animal/microbiome studies using V3–V4 Illumina and 16S rRNA gene MinION sequencing, Shin *et al*. (2016) investigated the mouse gut microbiome and found strong coherence at the order, family and genus levels for both approaches. However, bacterial species level discrimination was only possible using the MinION. Mitsuhashi *et al*. (2017) analyzed both a 20-member mock community and a clinical sample (effusion from a patient with a pleural cavity infection) using V2–V9 regions with the IonPGM system and 16S rRNA genes with the MinION. Their aim was to evaluate the suitability of a portable system using MinION for rapid clinical diagnosis. The authors found MinION sequencing could detect 91% of the bacteria in the mock community within 5 min (Fig. 2h in citation), although PCR and library prep took longer. Similar results between the IonPGM and MinION systems were observed despite large differences in analytical times. Yang *et al*. (2019) assayed endotracheal aspirate from 14 patients diagnosed with pneumonia and 8 control patients by sequencing genomic DNA on the MinION. They found high accuracy in pathogen detection for MinION with culture positive patients and could discern genetic information on antibiotic resistance. Along similar lines, Ibironke *et al*. (2020) examined the bacterial component in four compartments in the human respiratory system (lung [via lavage], throat, mouth and nose) from five subjects. The aim was to delineate the microbes that colonize the lungs, rather than being passively transported via inhalation. By comparing the quantitative signal within each compartment, it was possible to determine those microbes originating from within the lungs rather than being mobilized from the upper respiratory tract. Less than 5% of the OTUs detected throughout the respiratory tract were found to be enriched within lung samples.

Other MinION studies have focused on characterizing the microbiomes from freshwater, wastewater treatment or stormwater systems. For example, Acharya *et al*. (2019) looked at drinking water from 13 sites within the Kathmandu Valley. The focus was mostly on comparing qPCR with Illumina and MinION results for enteric bacteria and coliforms. The authors found, ‘significant rank correlations between the relative abundances of *Bacteroides*, *Prevotella*, *Enterobacteriaceae* and all other putative pathogenic genera determined by MinION and Illumina’. They concluded the MinION approach is a valid alternative to traditional methods for water quality monitoring. Likewise, Hamner *et al*. (2019) sequenced genomic DNA samples from a swimming hole in the Little Bighorn River and detected numerous pathogens in agreement with culturing efforts and could also discern antibiotic resistance genes (ARGs), while Reddington *et al*. (2020) sequenced genomic DNA from 11 riverine samples collected from Europe, North America and New Zealand. The authors could detect a dominant core microbiome containing 15 bacterial families and genes reflecting anthropogenic disturbance including hydrocarbon degradation and ARGs. In another study of ∼500 multidrug resistant isolates from 3 wastewater treatment plants in Hong Kong, Che *et al*. (2019) found nearly 1800 ARGs mostly associated with mobile elements and 16 different bacteria. The bulk of the ARGs (∼80%) were identified in members of the ESKAPE panel of pathogens (*Enterococcus faecium*, *Klebsiella pneumoniae*, *Acinetobacter baumannii* and *Pseudomonas aeruginosa*). Utilizing both short- and long-read approaches for the analysis, the authors conclude it is possible to comprehensively profile the genetic context of antibiotic resistance genes as well as to track their hosts across the wastewater treatment process. In a different study of stormwater/wastewater source tracking in Stockholm, a comparison of *E. coli* culture methods, Illumina V4 sequencing and MinION whole-genome sequencing found all approaches could successfully identify places where waste lines were misconnected with the stormwater system (Hu *et al*. 2018). The authors indicated MinION to be a rapid alternative to short-read approaches and had the potential to be utilized in the field. In a similar shotgun metagenome study of an enrichment bioreactor targeting phosphate retention, Arumugam *et al*. (2019) were able to generate six high-quality, circular genomes from a single MinION run. Using 4 µg of size-selected DNA (8 kb), the authors obtained 384× coverage of *Candidatus Accumilibacter*sp. SK-02, a polyphosphate accumulating microorganism commonly found in wastewater treatment plants. Circular genomes could also be generated from members of the *Bacteroidetes*, *Chloroflexi*, *Rhodospiralles* and *Chlamydia* groups with 10–60× coverage. The authors conclude that generation of whole bacterial chromosomes from complex, environmental samples will become routine.

Comparable MinION sequencing efforts have been applied to marine samples. Curren *et al*. (2019) investigated the marine cyanobacterial community and associated bacteria that could be grown in f/2 media from seven different sampling sites around Singapore and Malaysia. The authors could observe differences in cyanobacterial communities associated with the various sampling sites, while Hatfield *et al*. (2020) assayed two coastal samples experiencing a *Dinophysis* or *Alexandrium* bloom. The authors analyzed the 18S rRNA genes and the ITS region as a means of distinguishing the various dinoflagellate species within the harmful algal blooms. In a study of polar sediments, Millan-Aguinaga *et al*. (2019) performed shotgun genome sequencing on upper and lower samples from a core obtained in the Arctic and Antarctic using the MinION. Few of the reads were from bacteria (<5%) and mostly contained sequences from dinoflagellates or diatoms. Of those bacterial reads that could be identified, most were identified at the phylum level and included genes involved in processing of genetic and environmental information. The authors conclude the genomic approach was promising, but their difficulty in retrieving high-quality DNA from polar sediments prevented a more robust analysis. Additional efforts have been made to document the microbiome associated with other man-made structures using MInION. For example, Sheahan *et al*. (2019) described pathogen detection in emergency medical service vehicles with the MinION. The authors describe how pathogens can be detected in <24 h from various sites within the ambulance. Their results indicate, ‘there is a high likelihood that ambulances are indeed vehicles for pathogens into hospitals and vice versa’. Finally, Pinar *et al*. (2020) investigated the microbial community on two 18th–19th century oil paintings. Whole-genome amplification of DNA from multiple swabs collected from the painting surfaces indicated *Aspergillus fumigatus*, *Aspergillus glaucus* and *Cryobacterium arcticum* as major colonizers on one painting and *Cryobacterium arcticum*,*Ralstonia pickettii* and *Mycobacterium haemophilum* as colonizing the other. This study demonstrates how MinION can provide DNA sequence information related to preservation history of cultural heritage objects and could lead to a better understanding of those microbes responsible for bio-deterioration.

## FUTURE OUTLOOK

The MinION system has the potential to significantly change how we use sequence data for microbial ecology research. One example involves novel bacterial species/strain discovery as illustrated by Dowden *et al*. (2020) in the mouse gut microbiome. In this study of host genotype and exercise status, rRNA operons from many of the gut microbes were found to cluster based on experimental treatment. Long-read consensus reconstruction and phylogenetic analysis of those rRNA operons demonstrated bacterial species/strain level selection by the host, based on physical activity. This approach provides a tangible marker for differentiating isolates to improve culturing efforts and begin assessing physiological differences within strains that allow for selection by the host. Another major breakthrough with the MinION concerns RNA sequencing with nanopore technology (Harel *et al*. 2019; Smith *et al*. 2019). Direct RNA sequencing eliminates the need for reverse transcription and promises to be a more accurate assessment of gene expression. Likewise, nanopore sequencing can be used to determine base modification in DNA (Schreiber *et al*. 2013). Both Rand *et al*. (2017) and Simpson *et al*. (2017) demonstrated 95%+ accuracy in calling methylated cytosines in genomic DNA, which should make epigenetic studies easier and more routine. Another disruptive technology regarding MinION and real-time data analysis is the ability to analyze and potentially reject DNA strands within the nanopore during the run. Loose, Malla and Stout (2016), with their collaborators at ONT, have pioneered this technique that is called ‘Read Until’. The method could eliminate the need to deplete a sample of contaminating DNA (i.e. physically removing human DNA from a prep to profile the human microbiome), can balance reads between barcodes and may allow for specific detection of a target (e.g. a pathogen, or a specific region of a chromosome) in the shortest time possible rather than within a set time period. This ‘Read Until’ capability has been incorporated into a sequence-based, rather than squiggle space, platform called RUBRIC (Edwards *et al*. 2019). The authors state, ‘RUBRIC is specifically designed to function with … more modest computing resources …rather than high-end multiprocessor workstations or cluster computing platforms’. A similar sequence-oriented ‘Read Until’ may become part of the MinKNOW software in the near future and seamlessly be available to the MinION community. Lastly, coupling high-resolution microbiome profiling using the MinION with assays such as stable isotope probing or metabolome analysis could reveal the ways in which bacterial species/strains compete for resources in the environment or delineate the molecular mechanisms through which microbes interact with host organisms.

## CONCLUSION

It is clear that major improvements for nanopore sequencing have been made over the last 5 years in terms of accuracy and data analysis. Furthermore, it is clear the MinION is a serious sequencer, whose small size, low cost and ease of use belie the power of the platform. What is not clear is how this technology will ultimately be utilized when many more laboratories have access to a DNA/RNA sequencer as capable as any machine on the market. It should be very interesting to see what the next 5 years have in store for portable sequence analysis.
